# Smart Contract Vulnerability Detection Model Based on Multi-Task Learning

**DOI:** 10.3390/s22051829

**Published:** 2022-02-25

**Authors:** Jing Huang, Kuo Zhou, Ao Xiong, Dongmeng Li

**Affiliations:** 1 Faculty of Information Technology, Beijing University of Technology, Beijing 100124, China; zhoukuo@emails.bjut.edu.cn (K.Z.); lidm@emails.bjut.edu.cn (D.L.); 2 Beijing Key Laboratory of Computational Intelligence and Intelligence System, Beijing 100124, China; 3 State Key Laboratory of Networking and Switching Technology, Beijing University of Posts and Telecommunications, Beijing 100876, China; xiongao@bupt.edu.cn

**Keywords:** smart contract, security, vulnerability detection, multi-task learning

## Abstract

The key issue in the field of smart contract security is efficient and rapid vulnerability detection in smart contracts. Most of the existing detection methods can only detect the presence of vulnerabilities in the contract and can hardly identify their type. Furthermore, they have poor scalability. To resolve these issues, in this study, we developed a smart contract vulnerability detection model based on multi-task learning. By setting auxiliary tasks to learn more directional vulnerability features, the detection capability of the model was improved to realize the detection and recognition of vulnerabilities. The model is based on a hard-sharing design, which consists of two parts. First, the bottom sharing layer is mainly used to learn the semantic information of the input contract. The text representation is first transformed into a new vector by word and positional embedding, and then the neural network, based on an attention mechanism, is used to learn and extract the feature vector of the contract. Second, the task-specific layer is mainly employed to realize the functions of each task. A classical convolutional neural network was used to construct a classification model for each task that learns and extracts features from the shared layer for training to achieve their respective task objectives. The experimental results show that the model can better identify the types of vulnerabilities after adding the auxiliary vulnerability detection task. This model realizes the detection of vulnerabilities and recognizes three types of vulnerabilities. The multi-task model was observed to perform better and is less expensive than a single-task model in terms of time, computation, and storage.

## 1. Introduction

Smart contracting [1], a computerized trading protocol that codes paper contracts and executes terms, has lagged behind theoretical research in its application practice owing to the lack of a trusted execution environment. In 2008, Satoshi Nakamoto proposed a peer-to-peer (P2P) cryptocurrency system Bitcoin [2], whose underlying blockchain technology has decentralized characteristics and provides a reliable execution environment for smart contracts. Since 2013, the emergence of blockchain-driven, Turing-complete application platforms (Ethereum [3], Hyperledger [4], etc.) has provided opportunities for the development of smart contracts, resulting in a sharp rise in the number of contracts. However, as an emerging technology, smart contracts have a few defects in their programmable language and execution systems [5]. First, developers use high-level languages to code smart contracts and implement various complex business logic. However, these high-level languages (e.g., Solidity [6]) are extremely error-prone and contain programming errors that may be exploited and which cannot be detected until they are deployed on the blockchain. Second, smart contracts typically store and manage large amounts of financial assets and are operated on an open network, where any user can join and view the contract without a trusted third party, which increases the risk of the contract being attacked and can, in turn, bring substantial losses. Table 1 summarizes various attacks on smart contracts in recent years, each of which resulted in significant economic losses. In addition, because they run on distributed nodes on a blockchain platform, smart contracts cannot be modified after being deployed on a blockchain platform. Therefore, the vulnerability detection [7] of smart contracts is essential prior to their deployment on a blockchain platform. However, as most contract source codes are difficult to obtain, it is not practical to detect vulnerabilities only through smart contract source codes or to manually check whether the contract has vulnerabilities and identify their type.

To resolve the security issues concomitant to smart contracts, researchers have developed various detection tools that can detect the existing vulnerabilities. According to the technology employed, there are four main methods—symbolic analysis [11,12,13,14,15], formal verification [16,17,18,19,20,21,22,23], fuzzy testing [24,25,26,27], and other technologies [28,29,30,31]. Symbolic analysis is the most widely employed method and involves converting the uncertain input into a symbolic value in the process of program execution to promote the program execution and analysis. Symbolic execution can achieve a more accurate and comprehensive analysis of the program; however, it is typically confronted with problems such as path explosion due to the influence of program branches and loops [32]. Formal verification primarily employs rigorous demonstrable descriptive language or logic to describe the attributes and characteristics of the program and constructs formal specifications using mathematical logical proof and reasoning to determine whether the security attribute is set in line with expectations. However, formal verification entails a relatively low degree of automation and detects vulnerabilities that do not practically exist [33]. Fuzzy testing uses randomly generated test samples to trigger the vulnerability of the program and monitors the program’s abnormal behavior during its execution [34]. This method can effectively detect smart contract vulnerabilities, but its precision is low, and it cannot detect contracts without source code and call interface information. Other technologies mainly include program analysis and taint analysis, which are also widely used in program vulnerability mining and have a low cost but high false positive rate.

In recent years, machine learning (ML) methods used for program analysis have become a new trend in the field of security detection, which has a high degree of automation and breaks through the limitations of rule-based vulnerability detection of the existing detection methods. ML-based methods can extract hidden features from massive data (not limited to a single information feature) and have good scalability, which has caused researchers to introduce ML into smart contract vulnerability detection to achieve meaningful results [35,36,37,38,39]. However, the existing ML-based smart contract detection methods still have a few shortcomings—(1) they can only distinguish between leaky and non-leaky contracts (binary classification problem); (2) the source code of the smart contract is required, limiting its applicability; (3) it is not scalable and can only detect a few specific vulnerability types and cannot be extended to other vulnerability types.

To resolve the above-mentioned issues, this paper developed a smart contract vulnerability detection model based on multi-task learning [40], a new ML method, which can combine multiple learning tasks to complete multi-label classification and improve the accuracy and generalization ability of the model. By setting auxiliary tasks to learn more directional vulnerability features, the model can improve the detection effect and realize the detection and recognition of vulnerabilities.

The contributions of this paper are enumerated as follows:

- This paper proposes a smart contract vulnerability detection model and introduces multi-task learning into security detection. The model consists of two parts—(1) the bottom sharing layer, which uses neural networks based on the attention mechanism to learn the semantic information of input contracts and extract feature vectors; (2) the specific task layer, which uses the classical convolutional neural network (CNN) [41] to establish a classification network for each task branch. The captured features are learned from the sharing layer for detection and recognition to realize the detection of various vulnerabilities;
- The proposed multi-task learning model can effectively improve the precision of vulnerability detection in comparison with other methods. Compared to the single-task model, the multi-task model can complete multiple tasks at the same time, thereby saving costs in terms of time, computation, and storage. At the same time, this model can be extended to support the learning and detection of new vulnerabilities;
- In this study, we collected and downloaded 149,363 smart contracts running on real-world Ethereum from the XBlock platform [42] and used the existing detection tools to detect and label them and construct an open-source dataset containing the labeling information. This dataset provides key attributes, including the address, bytecode, and source code, and can be employed for smart contract vulnerability detection research.

The remainder of this paper is organized as follows: In Section 2, the paper provides the necessary background information on smart contracts and their vulnerabilities and briefly describes the pertinent literature. In Section 3, the design of the smart contract vulnerability detection model based on multi-task learning is introduced in detail, and in Section 4, the experimental details and evaluation results are described in detail. Finally, Section 5 includes the discussion and conclusion of this paper.

## 2. Background and Related Work

### 2.1. Smart Contracts and Vulnerability

**Figure 1:**
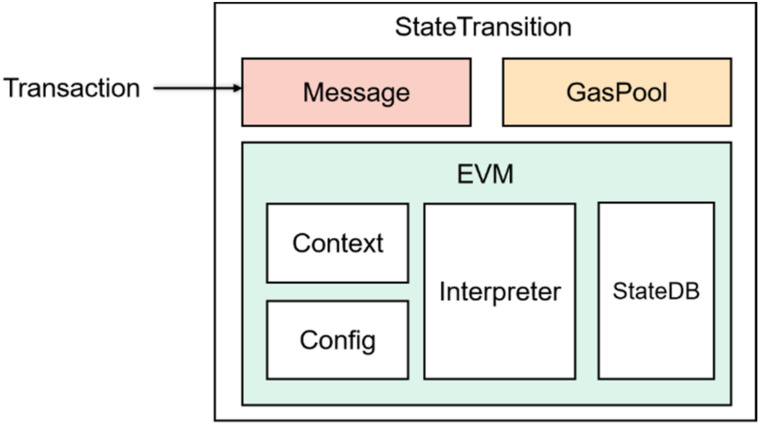
EVM business process.

In this study, the authors used the smart contracts running on Ethereum [3] as the research object. Ethereum, one of the most popular blockchain platforms, provides a decentralized, Turing-complete Ethereum Virtual Machine (EVM) [43] to handle the execution and invocation of smart contracts through its dedicated cryptocurrency. EVM is a stack-based machine with a 256-bit word size and a maximum stack size of 1024 for performing transactions on Ethereum [16]. The business process is depicted in Figure 1. When a transaction is entered, it is internally converted to a message, which is then passed to EVM for execution. When the smart contract is invoked, the bytecode is loaded and executed through the interpreter in EVM. Unlike traditional applications, smart contract execution and transactions require the gas to be maintained, which is the unit used to pay miners the computational cost of running the contract or transaction paid in cryptocurrency [44].

As a kind of code, smart contracts are compiled by EVM to generate bytecode and executed on a blockchain platform through address invocation. As mentioned above, smart contracts have defects in their programming languages and execution systems. Researchers have collected this vulnerability information and created the Smart Contract Vulnerability Classification (SWC) Registry [45]. Considering the economic loss and harm caused by contract vulnerabilities to the real world, three classic and common vulnerabilities for detection have been selected, namely arithmetic vulnerability (SWC-101 [45]), reentrancy (SWC-107 [45]), and the contract contains unknown address. They are described as follows.

(1) Arithmetic vulnerability: This type of vulnerability is also known as integer overflow or underflow, arithmetic problems, and so forth. It is very common because programming languages have a length limit for integer types of storage, and it occurs when the results run outside of this range. For example, if a number is stored in the uint8 type, it means that the number is stored in an 8-bit unsigned number ranging from 0 to 255, and an arithmetic vulnerability occurs when an arithmetic operation tries to create a number outside of that range. Arithmetic vulnerability is also one of the most common vulnerabilities in smart contracts, and malicious attackers use this vulnerability to steal a large number of tokens, resulting in considerable economic losses.
(2) Reentrancy: The ability to call external contract codes is one of the features of smart contracts, and contracts can send digital currency to external user addresses for transactions. Such calls to external contracts may cause reentrancy. The attacker uses reentrancy vulnerability to perform the recursive callback of the main function and continuously carries out the “withdrawal” operation in the contract until the account balance in the contract is cleared or the gas upper limit is reached. In 2016, the DAO attack [8] took place, wherein a malicious attacker applied to the DAO contract for funds several times before the contract balance was updated. The vulnerability was caused by a code error in which the developer failed to consider recursive calls. Although Ethereum resolved the attack with a blockchain hard fork, it still caused significant economic losses.
(3) Contract contains unknown address: Smart contracts are P2P computer transaction protocols. Thus, when a contract contains an unknown address, this address is likely to be used for some malicious activities. When this vulnerability occurs, it is required to check the address. In addition, it is required to check the code of the called contract for vulnerabilities.

### 2.2. Methods for Smart Contract Vulnerability Detection

Automated vulnerability mining is an important area of software vulnerability mining. The authors of this paper examined the literature on five related types of research—symbolic execution, formal verification, fuzzy testing, other technologies, and ML-based methods.

(1) Symbolic execution: Oyente [11] was one of the earliest works to use symbolic execution for detecting vulnerabilities in the source code or bytecode of smart contracts. It constructs the control flow graph and uses it to crease inputs and provides a symbolic execution engine for other tools. Osiris [12] has been improved based on Oyente, using symbolic execution and taint analysis to detect vulnerabilities. Similarly, Manticore [15] analyzes contracts by executing symbolic transactions against the bytecode, tracking the contracts’ states, and verifying the contracts. Maian [14] and Mythril [13] are also based on symbolic execution.
(2) Formal Verification: Hirai et al. [46] used Isabelle/HOL to formalize contracts to prove their security. In addition, many methods have been developed to formalize smart contracts, such as ZEUS [18], which translates source codes into LLVM intermediate language, uses XACML to write validation rules, and then uses SeaHorn [47] to formalize validation. Securify [22] and VerX [23] are two other mainstream formal verification tools.
(3) Fuzzy Testing: Fuzzy testing has been widely used in the vulnerability mining of traditional programs, and it attempts to expose vulnerabilities by executing the program with inputs. Echidna [24], published by Trail of Bits, is a complete fuzzy testing framework for analyzing smart contracts and simulation testing. ContractFuzzer [25] is also a kind of fuzzy testing scheme that performs vulnerability detection by recording the instruction log during the execution of smart contracts. ILF [26] is a fuzzy testing scheme based on neural networks that are used to generate better test cases in fuzzy testing.
(4) Other technologies: Program analysis and taint analysis are also commonly used in vulnerability detection, and program analysis involves determining the safety of a program by analyzing it to obtain its characteristics, while taint analysis involves marking key data and tracking its flow in the process of program execution to achieve program analysis. SASC [28] is a smart contract vulnerability detection based on static program analysis methods that searches for control flow characteristics to detect vulnerabilities through automatic analysis of the source code. Similarly, SmartCheck [29] and Slither [30] are also detection tools based on program analysis. Sereum [31] uses taint analysis to trace data streams to detect vulnerabilities.
(5) ML-based methods: As mentioned above, the security of smart contracts has garnered public attention, and some achievements have been made in the research of contract vulnerability detection methods using ML. TonTon Hsien-De Huang [35] proposed a method for the in-depth analysis of potential vulnerabilities that involves converting bytecode into an RGB image and then training a CNN for automatic feature extraction and learning. Sun et al. [48] added an attention mechanism [49] to CNN to further improve its accuracy in detecting vulnerabilities. Wesley et al. [36] used a long short-term memory (LSTM) neural network to learn vulnerabilities by a sequential learning method and realized a relatively fast detection of vulnerability contracts. However, these methods can only be used to distinguish whether there are vulnerabilities, which are essentially binary classification models, and they cannot identify the types of vulnerabilities or detect multiple vulnerabilities. To realize the detection of multiple vulnerabilities, Moment et al. [50] and ContractWard [37] used various ML algorithms (support vector machine, decision tree, random forest, XGBoost, AdaBoost, k-nearest neighbor, etc.) to establish an independent classification model for each vulnerability; the main difference is that the former used an abstract syntax tree to construct the vulnerability features of a smart contract, while the latter used an N-Gram language model [51] to extract binary syntax features from the simplified opcodes of smart contracts. Although these two methods have realized various vulnerability detection, they are still dichotomous and not separate models. In addition, ESCORT [52] proposed a multi-output architecture that connected each vulnerability classification branch to the feature extractor based on a deep neural network (DNN) and established a separate output for each vulnerability type, thus realizing the detection of multiple vulnerabilities. This provided the preliminary idea for our model design, and in this work, we propose a multi-task learning-based model for smart contract detection, which not only detects the presence of vulnerabilities in the contract but also recognizes the types of contract vulnerabilities, that is, it includes multi-vulnerability detection.

### 2.3. Multi-Task Learning

Multi-task learning is usually only about optimizing for specific metrics in ML. To do that, a single model is trained to perform tasks to get the desired results, and then fine-tune models until the performance is at its best. Although this single-model approach often results in better performance, if one focuses only on a single indicator, they may miss some relevant information. This neglected indicator information can make the single-task model achieve better performance to some extent. In essence, there is some information from related tasks, and the shared representation between tasks enables the model to better summarize the original task, an approach known as multi-task learning [40].

The main purpose of multi-task learning is to jointly learn multiple related tasks such that the information contained in the task can be used by other tasks, and the learning efficiency and generalization ability of the model can be improved by sharing this information. With this method, smart contracts are treated as a special long sequence of text and they are learned from natural language processing (NLP) to process them. Multi-task learning has been widely used in the field of NLP and has had some achievements. Collobert et al. [53] proposed a CNN model based on multi-task learning to demonstrate the excellent performance of multi-task learning in natural language processing, including part-of-speech tagging, named entity recognition, and semantic role tagging. Niu et al. [54] proposed a character-level CNN model to normalize disease names based on multi-task learning and introduced an attention mechanism to optimize the model effect. Liu et al. [55] designed three information-sharing mechanisms based on LSTM and used the sharing layer of a specific task to model the text. Their study found that sub-tasks could improve the performance of the main classification task. Yang et al. [56] proposed an attention-based multi-task BiLSTM-CRF model with embeddings from language models (ELMo) as a vector, which further improved the entity recognition and normalization effect. In this current study, multi-task learning was incorporated into the task of smart contract vulnerability, as it can improve the performance of the main task by sharing knowledge. By setting auxiliary tasks, the proposed model can learn more directional vulnerability features, improve the detection effect, and realize the detection and recognition of vulnerabilities.

**Figure 2:**
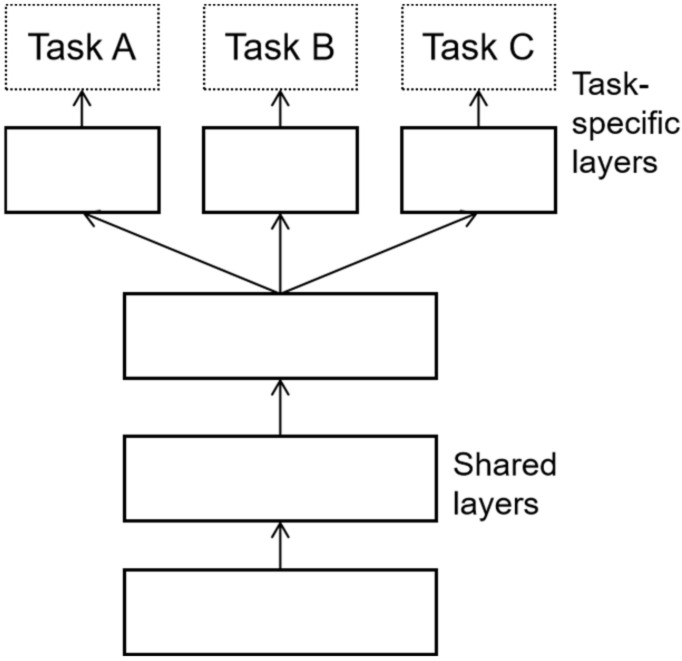
The hard parameter sharing method.

**Figure 3:**
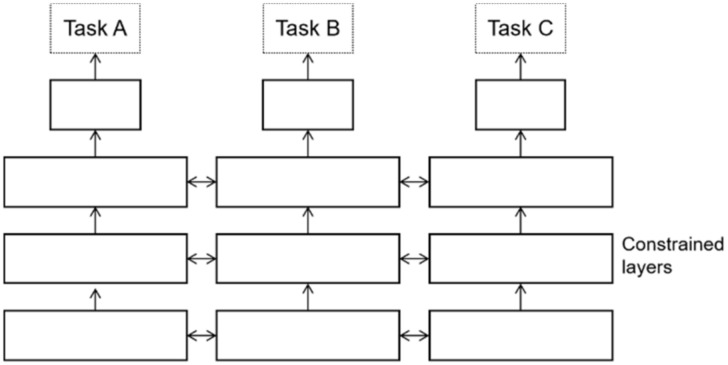
The soft parameter sharing method.

There are two types of multi-task learning models—the hard parameter sharing method [57] and the soft parameter sharing method [58]. The former method, illustrated in Figure 2, is the most common sharing strategy wherein different tasks share the model part except for the output layer, which can train the general representation of multiple tasks at the same time and effectively avoid the over-fitting risk caused by less training data. The latter method, depicted in Figure 3, does not share the parameter structure directly, and each task has its model and parameters. The parameter similarity of the model can be guaranteed by the regularization of the parameters of similar parts of the model. In this study, the authors proposed a multi-task learning framework based on the hard parameter sharing method, which shares the underlying parameters; the parameters of each model at the top are independent of each other to reduce the possibility of model overfitting.

## 3. The Proposed Smart Contract Vulnerability Detection Model

### 3.1. Data Collection and Preprocessing

#### 3.1.1. The Source Codes, Bytecodes, and Operation Codes of Smart Contracts

**Figure 4:**
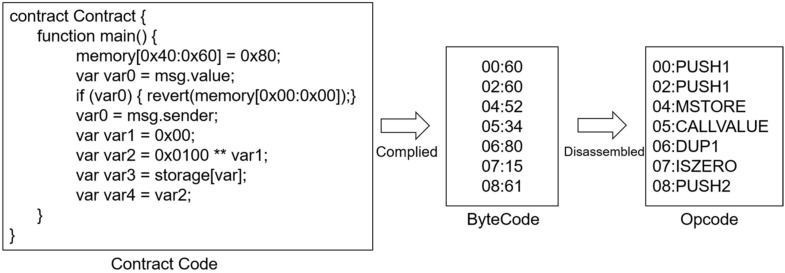
The compiling order among different code types of smart contracts. The three text frames from left to right represent the three code types, respectively. The leftmost frame represents the source codes of smart contracts, which are written in a high-level programming language, Solidity, where ** represents the exponential operator. The middle frame represents the byte codes of smart contracts, which are a cluster of hexadecimal digits corresponding to a specific operation code, for example, the bytecode 60 in the first line corresponds to the opcode PUSH1 in the first line of the rightmost frame, which represents the operation codes. When processing the smart contracts, the source codes are firstly compiled into byte codes, then into operation codes, that is, opcodes, which are used as data in the model.

Deploying a smart contract on a blockchain platform entails three steps. First, developers use a high-level language (e.g., Solidity [6]) to code the smart contracts; second, the source codes are compiled into bytecode with a compiler; third, the bytecodes are uploaded to EVM via an Ethereum client and translated into operation codes (opcodes). However, because contract source code is unavailable and there are many man-made variables defined in source codes, it is not appropriate to analyze smart contracts using source code. Furthermore, smart contracts run on blockchains in the form of bytecode, which is easier to obtain. The relationships among source codes, bytecodes, and opcodes of smart contracts are illustrated in Figure 4.

#### 3.1.2. Data Acquisition

In this study, we collected and downloaded 149,363 smart contracts running on real-world Ethereum from the XBlock platform, which contains nine contract attributes, namely the address, contract code, timestamp, create value, create block number, created transaction hash, creation code, creator, and code [42]. Next, the data were cleaned by writing data-cleaning scripts to remove redundant, repeated, invalid, and vacant data. We then used a vulnerability detection tool to label all source code files and obtain the labeled data, including whether the contracts have vulnerabilities (Flag) and the specific types of vulnerabilities (Label). Specifically, each contract has four tags, and the tags are independent of each other for each type of vulnerability. Each label consists of a four-dimensional column [$x_{1},x_{2},x_{3},x_{4}$], and each element $x_{i}\left(i=1,2,3,4\right)$ has a value of 0 or 1. For $x_{i}=1\left(i\neq 1\right)$, the smart contract has the i-th vulnerability; otherwise, there is no such vulnerability. In particular, $x_{1}$ represents the label “Flag”, which indicates whether the smart contract has any vulnerabilities. Next, the labels were assumed to be correct. Finally, the datasets constructed in this study contain 141,387 smart contracts and four kinds of tag information. A few samples of the smart contracts datasets are presented in Table 2.

Bytecodes are byte arrays encoded by hexadecimal digits, which represent a specific sequence of operations and parameters; however, they take up a large amount of memory space to analyze and model long sequences of bytecodes. It is thus impractical to directly use bytecode as model input. The bytecode representation of smart contracts has a one-to-one mapping relationship with the opcode of blockchain, and thus, it is feasible to detect contract vulnerabilities at the opcode level. Therefore, it is necessary to convert the bytecode to generate the opcode; the opcode was used as data in this research.

According to Ethereum Yellow Paper [16], there are 142 operation instructions with 10 functions, including arithmetic operations, bit-wise logic operations, block information operation, comparison, stack, memory, storage, jump instruction, and so forth. If all these instructions are used, it may lead to a dimension disaster caused by the presence of too many instructions. Fortunately, some operating instructions have little to do with the behavior of the source code, and thus, the role of these instructions can be neglected in the process of vulnerability detection [48]. Likewise, the opcodes are simplified by classifying opcodes with similar functions; specifically, each push instruction is followed by an operand that can be removed [37]. The simplified opcode methods are shown in Table 3.

The sample data of simplified opcodes is shown in Table 4.

#### 3.1.3. Data Imbalance

As a typical anomaly detection problem, the number of smart contracts with vulnerabilities is far lower than the number of normal contracts, and the number of contracts with specific vulnerabilities is even lower. Thus, it is essentially an imbalanced classification problem. Most of the current methods to solve data imbalance are resampling, including under- and over-sampling [59], the main idea of which is to reduce the influence of imbalanced data on the classifier by adjusting the proportion of imbalanced data. In particular, the under-sampling method balances data by reducing the number of samples from most classes, while the over-sampling method improves the classification performance by increasing the number of samples from a few classes.

**Figure 5:**
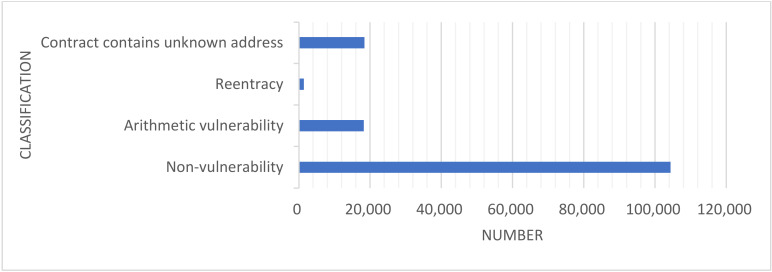
The number of vulnerability categories in the original datasets. Most of the contracts have non-vulnerability and the least number of contracts have reentrancy.

**Figure 6:**
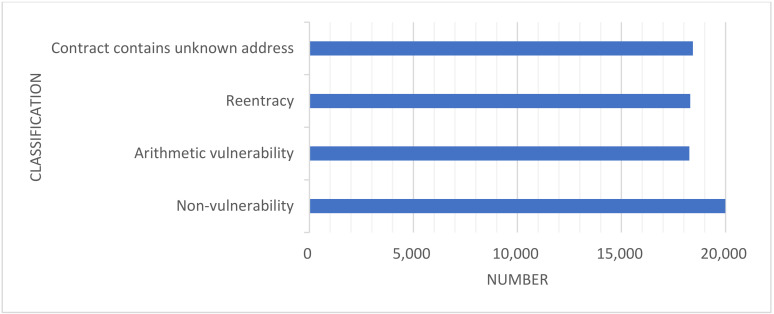
The number of vulnerability categories after balancing datasets. The numbers of contracts with various vulnerabilities are almost the same.

As depicted in Figure 5, the number of category vulnerabilities in the original dataset is highly unbalanced. Therefore, resampling this dataset includes the under-sampling of contracts without vulnerabilities, thereby reducing the number of samples of most classes. It also includes the over-sampling of contracts with vulnerabilities, increasing the number of samples of a few classes to achieve balance in the dataset. Table 5 shows the number of different contracts before and after sampling, and the balanced dataset is shown in Figure 6.

### 3.2. Model Design

**Figure 7:**
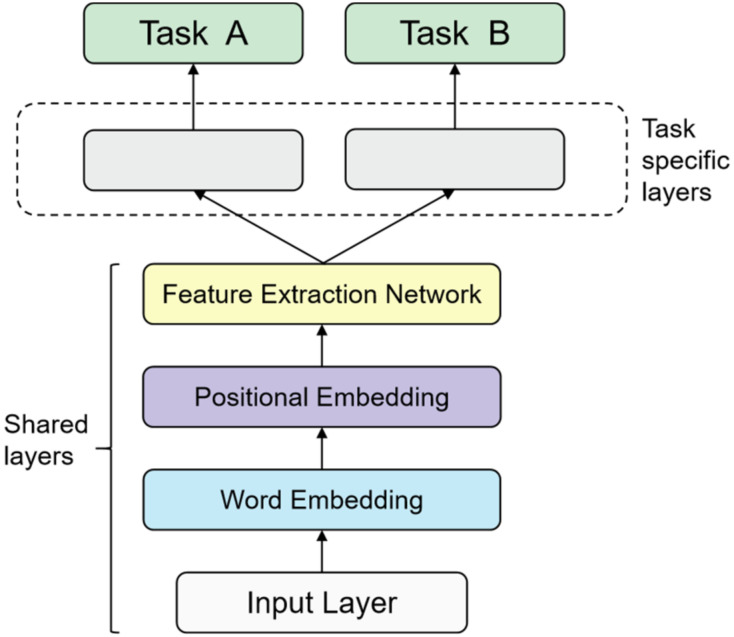
Smart contract vulnerability detection framework based on multi-task learning.

The paper developed a smart contract vulnerability detection model based on multi-task learning; the model framework is shown in Figure 7. An efficient multi-task network structure must take the shared part and the specific part at the same time into account. It not only needs to learn the generalized representation between different tasks to avoid over-fitting but also needs to learn the unique characteristics of tasks to avoid under-fitting [40]. Therefore, the model is mainly divided into two parts—the bottom sharing layer and the top specific tasks layer.

#### 3.2.1. The Bottom Sharing Layer

The design of the bottom sharing layer is used to determine how to achieve knowledge sharing among all tasks in the multi-task learning model. In multi-task learning, the shared content mainly includes features, instances, and parameters. Feature-based multi-task learning is mainly used to learn common features among different tasks. Instance-based multi-task learning mainly attempts to identify useful data instances in one task for other tasks and then share knowledge through the identified instances. Parameter-based multi-task learning uses model parameters of one task to help learn model parameters in other tasks [40]. As mentioned above, the opcode of smart contracts is regarded as a special long-sequence text and use the knowledge of NLP to convert the text representation into a digital vector representation and then extract text features for feature learning to complete specific tasks. In this study, we, therefore, chose to implement a multi-task learning model by sharing features.

**Figure 8:**
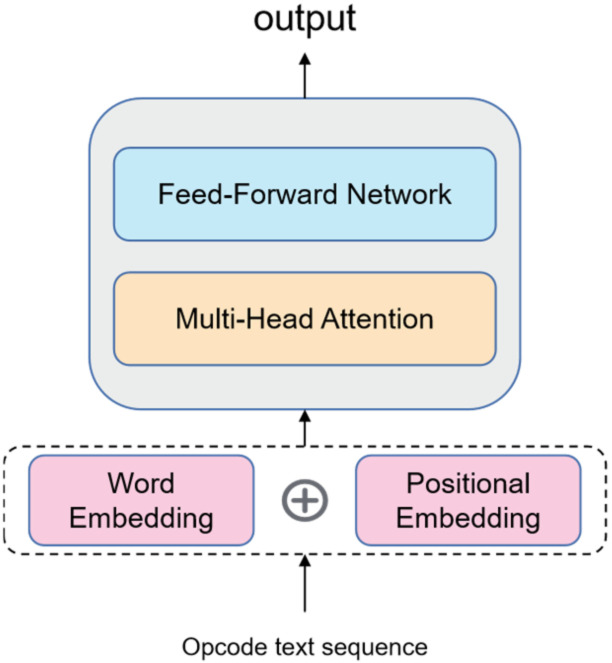
The bottom shared layer design framework, including word embedding, positional embedding, multi-head attention layer, and feed-forward network.

In addition to sharing knowledge, another problem with the design of the sharing layer is how to share it, which refers to the specific ways in which knowledge is shared between tasks. In feature-based multi-task learning, knowledge sharing between tasks is realized mainly by learning key features. Existing studies have shown that the attention mechanism can make the neural network focus on key features. Therefore, the proposed model constructs a sharing layer network based on the attention mechanism to enable the model to learn the features of smart contract sequences; its architecture is depicted in Figure 8.

The bottom shared layer uses word embedding and positional embedding to describe the smart contract opcode sequence. Word embedding converts each input opcode into the form of a word vector to map the opcode sequence into a multi-dimensional space. To make the model understand the order of the opcode sequence, positional embedding uses the position information of words to make a secondary representation of each word in the sequence. Adding the position vector to the word coding combines the word order information with the word vector to form a new representation in the subsequent calculation, which can better express the distance between words and complete the description of the input sequence. Therefore, the proposed model can learn the information of the opcode sequence. The specific calculation formula is as follows:

(1) PEpos,2i=sinpos10,0002id

(2) PEpos,2i+1=cospos10,0002id

Using sinusoidal position encoding makes it easy for the model to learn to care about relative position information, so for any fixed offset i, $PE_{\left(pos+i\right)}$ can be expressed as a linear function of $PE_{\left(pos\right)}$. In the formula, pos represents the position of the word in the sequence, $PE_{\left(pos,2i\right)}$ and $PE_{\left(pos,2i+1\right)}$ represent the vector corresponding to the pos position, d represents the dimension of the vector, and 110,0002id represents the frequency $w_{k}$. Therefore, the positional vectors are a set of sine and cosine pairs that map each frequency.

After encoding by word embedding and positional embedding, the opcodes are input into a multi-head attention layer for learning and extracting features. When the model processes each word in the input sequence, the self-attention network of the multi-head attention layer [49] helps the encoders pay attention to all words in the whole input sequence and assists the model to view other positions in the input sequence to achieve a better encoding effect. The input consists of queries and keys of dimension $d_{k}$ and values of dimension $d_{v}$. The output of the self-attention networks can be computed as follows:

(3) $$Attention\left(Q,K,V\right)=softmax\left(\frac{QK^{T}}{\sqrt{d_{k}}}\right)V$$

where *Q*, *K*, *V* is a set of a query vector, key vector, and value vector formed by multiplying the input vector by three weight matrices, respectively. We computed the products of the query with all keys, divide each by $\sqrt{d_{k}}$, and applied a *softmax* function to obtain the weights of the values [49].

**Figure 9:**
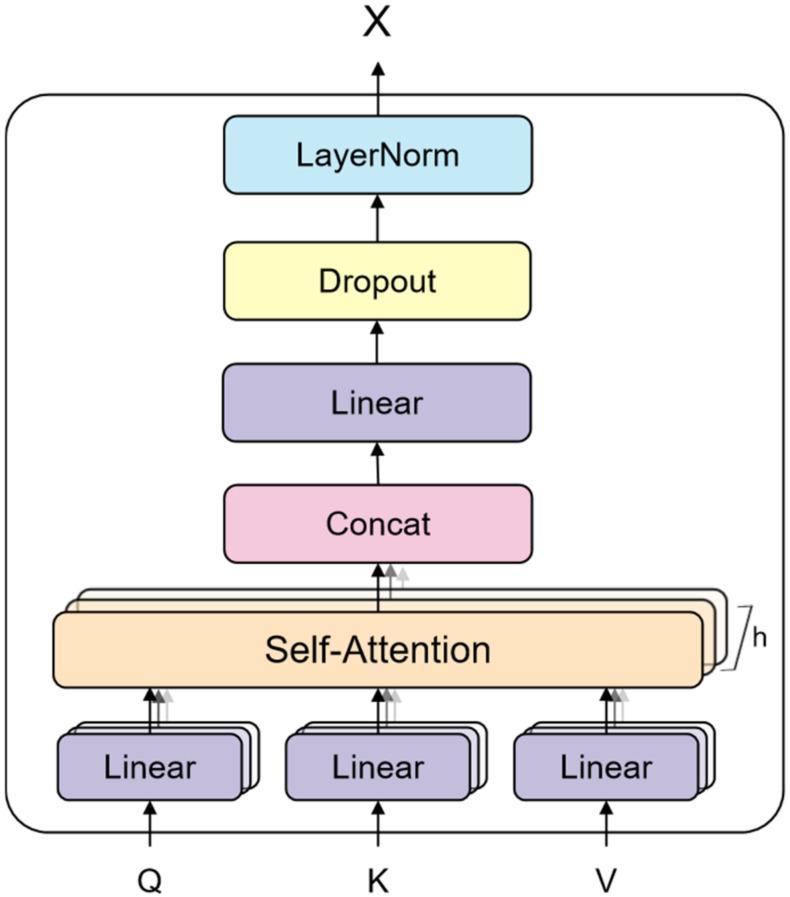
Multi-head attention network consisting of multiple self-attention layers—Concatenate layer, Linear layer, Dropout layer, and LayerNorm layer.

The multi-head attention layer performs the same self-attentional network calculation mentioned above. The difference is that each $head_{i}$ projects the input vectors *Q*, *K*, *V* through different linear transformations to maintain the independent vector weight matrices of each head, thus generating different attention vectors matrices. Finally, the vectors generated by each head are superimposed and spliced, resulting in a vector–matrix that incorporates all the attention head information. The multi-head attention network structure used by the model is shown in Figure 9; it improves the performance of the attention layer and expands the model’s ability to focus on different positions. The output of the multi-head attention layer is computed as follows:

(4) $$X=MultiHead\left(Q,K,V\right)=Concat\left(head_{1},\ldots ,head_{h}\right)W^{o}$$

(5) $$where,\;head_{i\;}=Attention\left(QW_{i}^{Q},KW_{i}^{K},VW{\;}_{i}^{V}\right)$$

**Figure 10:**
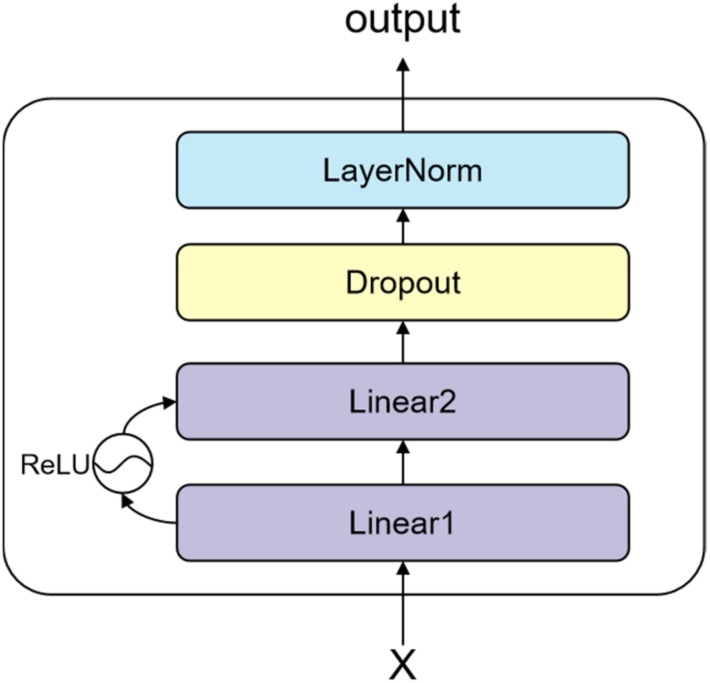
Feed-forward neural network consisting of Linear layers, Dropout layer, and LayerNorm layer.

The model sends the vector–matrix output from the multi-head attentional network to the feed-forward network and outputs the results of the feature-sharing layer. The network structure is depicted in Figure 10. The matrix of output can be computed as follows:

(6) $$Output=LayerNorm\left(X+\left(FFN\left(X\right)\right)\right)$$

#### 3.2.2. The Top Specific Tasks Layer

To implement the multi-task learning model, we had to determine what tasks the model needs to perform. This model is mainly used for the vulnerability detection of smart contracts, so tasks are divided into detection and recognition. For detection, which is essentially is a binary classification task, the classic CNN is used to build a binary classification network. The feature vector calculated by the shared layer is taken as the input of the convolution layer, and the important information in the feature vector is extracted by the CNN. Next, focal loss [60] is used to calculate the task loss. The specific formula is as follows:

(7) $$Loss_{fl}=-\frac{1}{n}\overset{n}{\underset{i=1}{\sum }}\alpha {\left(1-y_{i}^{\prime }\right)}^{\gamma }logy_{i}^{\prime }+\left(1-\alpha \right)y_{i}^{\prime }{}^{\gamma }\log\left(1-y_{i}^{\prime }\right)$$

Focal loss is modified in accordance with the cross-entropy loss function, primarily to resolve a serious imbalance in the proportion of positive and negative samples for target detection and to reduce the weight of a large number of simple negative samples in training. In the formula, $y^{\prime }$ is the probability that the sample i is predicted to be a positive class; γ is the adjustment factor, which is used to adjust the rate of the weight reduction of simple samples, pay special attention to samples that are difficult to distinguish, and to reduce the impact of simple samples. The loss is the cross-entropy function when γ=0; α is a balancing factor used to balance the proportion of and adjust the importance of positive and negative samples. In this study, along the lines of the paper [60], default values (γ=2 and α=0.25) were used.

The recognition task is essentially a multi-label classification task. As with the detection task, the classic CNN was still used to build the task network. The difference is that multiple neurons are added to the last fully connected layer of the branch network layer, and the probability output of each sample is calculated through the softmax function. Finally, the cross-entropy loss function was used to calculate this task loss. The recognition loss was calculated as follows:

(8) $$\begin{array}{c} Loss_{ml}=-\frac{1}{n}\sum_{i}\sum_{c=1}^{M}y_{ic}\log\left(p_{ic}\right) \end{array}$$

where *M* is the number of categories. $p_{ic}$ is the predicted probability that sample i belongs to category c, $y_{ic}$ is the sign function (0 or 1), and if the true category of sample i is equal to c, then $y_{ic}=1$, or $y_{ic}=0$.

**Figure 11:**
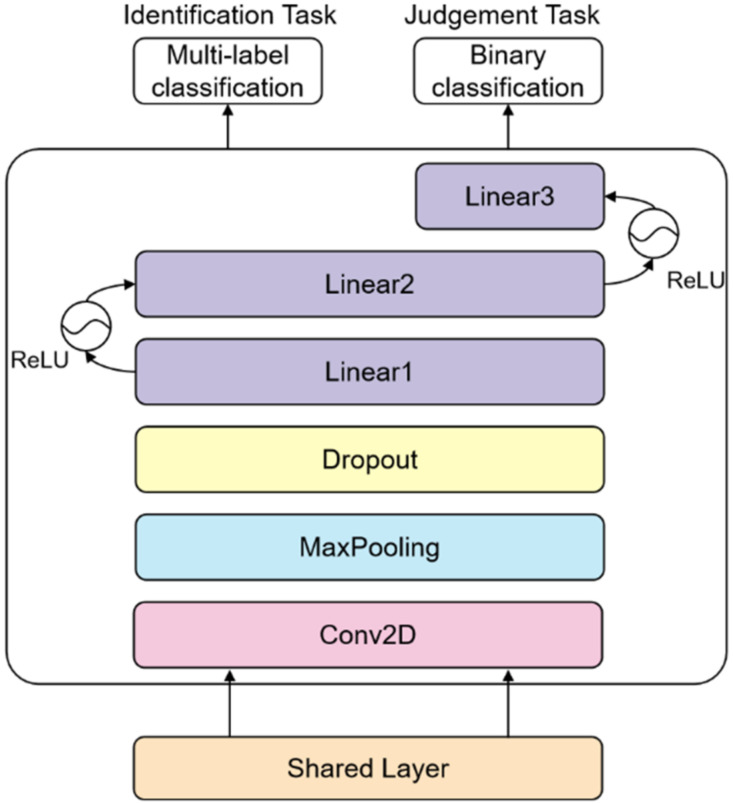
Task layer branch network, where multi-label classification is the identification task, binary classification is the detection task.

The design of the task-level branch network is depicted in Figure 11.

Multi-task learning is a method of derivation and transfer, the idea of which entails an ML-based method that puts multiple related tasks together to learn through shared representation. Therefore, whether there is a correlation between tasks is an important problem in model design. According to paper [40], if two tasks use the same features to make decisions, the tasks can be considered to be similar. The proposed model uses the same datasets, learns and obtains features from the datasets, and the detection and recognition tasks share the same features. Therefore, it is believed that the two tasks are related.

In addition, multiple related tasks share information and optimize parameters through the mutual adjustment of the loss function in multi-task learning, followed by feedback to each sub-task to improve the model effect. The sharing loss function was calculated as follows:

(9) $$\begin{array}{c} Loss=\frac{Loss_{fl}+Loss_{ml}}{2} \end{array}$$

The algorithm pseudocode flow is shown in Algorithm 1 as follows:
**Algorithm 1.** Training model.1: Initialize model parameters randomly 2: Pre-train the shared layers 3: Set the max number of epoch: $epoch_{max}$
4: for t in 1, 2, …, T do 5:   Pack the dataset t into mini-batch: $D_{t}$ 6: End 7: for epoch in 1, 2, … , $epoch_{max}$ do 8:   1. Merge all the datasets: 9:    D =$\;D_{1}\cup D_{2}\cup \ldots \cup D_{T}$
10:   2. Shuffle D 11:    for $b_{t}$ in D do // $b_{t}$ is a mini-batch of task t 12:     3. Compute loss: $\mathrm{Loss}\left(\theta \right)$
13:        $Loss_{fl}\left(\theta \right)$ = Equation (7) for judgment task 14:        $Loss_{ml}\left(\theta \right)$ = Equation (8) for identification task 15:        $Loss\left(\theta \right)$ = Equation (9) for multi-task learning model 16:     4. Compute gradient: $\nabla \left(\theta \right)$
17:     5. Update model: $\theta =\theta -\varepsilon \nabla \left(\theta \right)$
18:    End 19: End

## 4. Experiment and Analysis

To verify the efficacy of our model, in this study, we conducted four groups of experiments. Experiment 1 was a multi-task learning experiment that tested different parameters and carried out a vulnerability detection experiment. The remaining experiments were comparative. In Experiment 2, the model was compared with existing vulnerability detection tools to verify the detection effect of the model compared with the benchmark method. In Experiment 3, the model was compared with other ML-based vulnerability detection, and Experiment 4 was an ablation experiment. Each experimental dataset was divided into a training set, validation set, and test set at a ratio of 7:2:1.

### 4.1. Experimental Setup

All the experiments were carried out under the settings listed in Table 6. In addition, the Jieba word segmentation database, word2vec, and Glove word vector training tools were also used in the experiment.

### 4.2. Evaluation Metrics

We evaluated the performance of our model in terms of precision, recall, and $F1_{score}$; each metric is detailed as follows.

The results of true positives (*TP*), true negatives (*TN*), false positives (*FP*), and false negatives (*FN*) are the basis for calculating other metrics, where true values represent the number of correctly predicted results, whereas false values represent the number of wrongly predicted results [61]. The precision metric describes the proportion of correct predictions to all correct predictions, indicating the accuracy of the classifier’s positive prediction [62]. The recall metric represents the proportion of actual positives that are correctly classified. The formulas to compute these two metrics are given as follows:

(10) $$Precision=\frac{TP}{TP+FP}$$

(11) $$Recall=\frac{TP}{TP+FN}$$

The $F1_{score}$ metric is also a commonly used measurement index that uses precision and recall for quantifying the accuracy of the overall decision. This metric is defined as the harmonic average of precision and recall, and its calculation formula is as follows:

(12) $$F1_{score}=\frac{2*Precision*Recall}{Precision+Recall}$$

### 4.3. Experiments

#### 4.3.1. Experiment 1: Multi-Task Learning Model Experiments

Experiment 1 was an investigation into the influence of parameters on detection performance. Owing to the large number and wide range of variables, it was difficult to select the optimal value for each combination of variables. Therefore, the values of some variables were changed while determining the other variables to find a relatively good combination of parameters.

This model involves multiple parameters (see Table 7). In addition, this experiment involved the setting of the neural network training parameters, including the epoch and batch size. Based on experience, the experiment initially set a batch size of 32 and an epoch of 150, focusing on the training accuracy changes as the training process converged.

**Figure 12:**
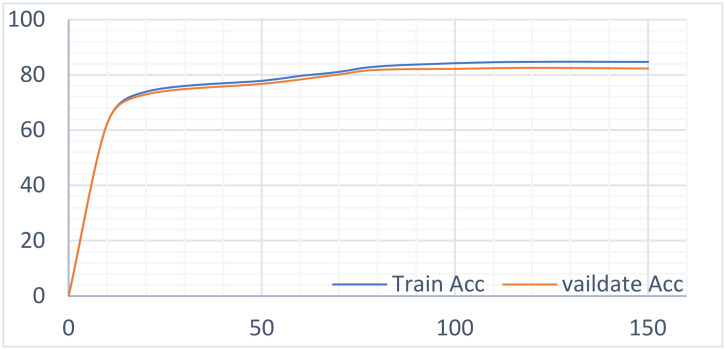
Training accuracy of different epochs.

As is evident in Figure 12, the training precision and the test precision increased with the increase in the epoch at first. When the epoch reached 100, the precision began to stabilize. Therefore, the initial value of the epoch was set to 100 to achieve high precision without making the training time too long.

**Figure 13:**
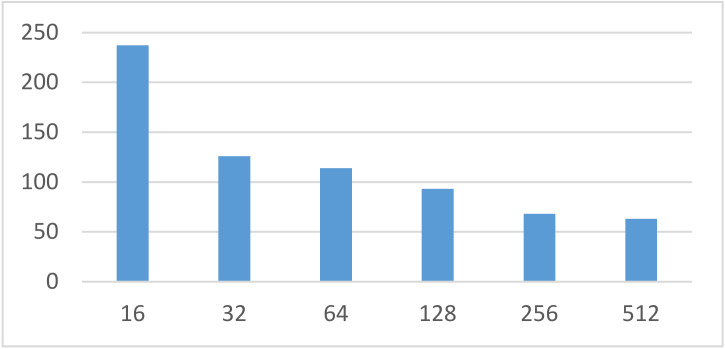
Training times of different batch sizes.

**Figure 14:**
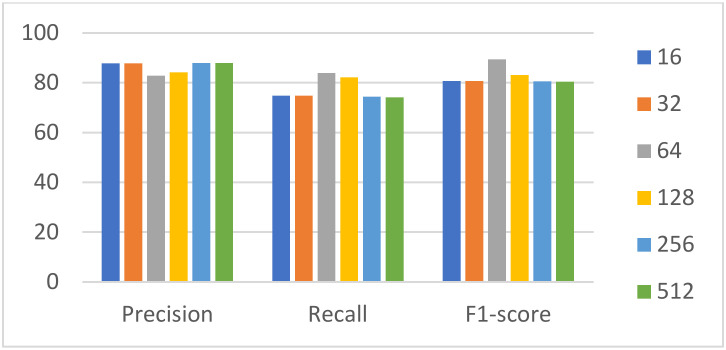
Training metrics for different batch sizes.

Batch size is related to the gradient descent direction of the model in each learning process. A batch size that is too large or too small affects the training time and model accuracy. The epoch was set to 100, and the batch size incrementally increased within {16,32,64,128,256,512}. Figure 13 and Figure 14 represent the time required for the model to complete the training and the training index.

From Figure 13, it is evident that as the batch size increased, the training time decreased. When the batch size was greater than 64, the time spent in training did not change significantly. Furthermore, the training metrics fluctuated within a certain range with the increase in batch size. Precision decreased first and then increased; recall and $F1_{score}$ increased first and then decreased, reaching the highest value when the batch size was 64. By weighing the time required for the training model against the training metrics, the batch size was finally set to 64. The parameter settings used in this experiment are shown in Table 7.

**Figure 15:**
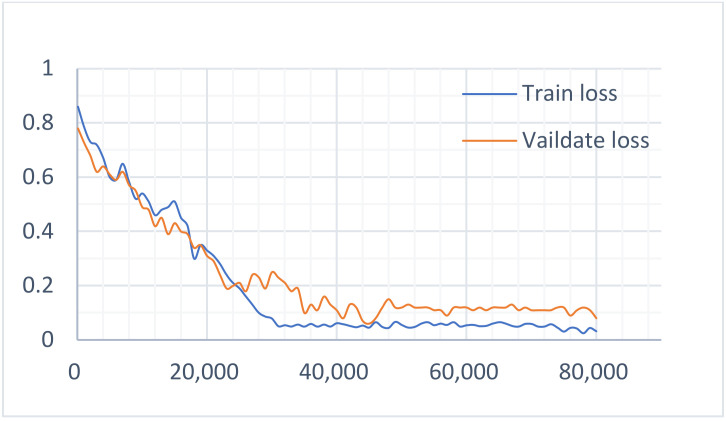
The loss curve of the detection task.

**Figure 16:**
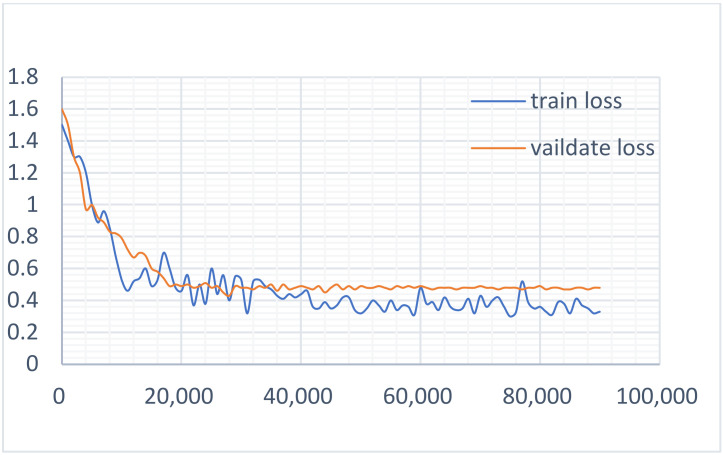
The loss curve of the recognition task.

**Figure 17:**
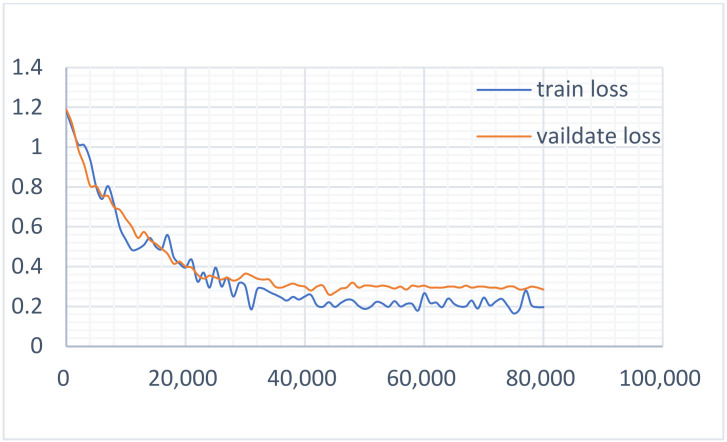
The loss curve of the multi-task learning model.

According to the above-mentioned parameter settings, we completed the multi-task learning experiment on the constructed datasets. To determine the influence of the two main tasks on the model, the loss value curves of the two tasks in the training and verification of the dataset were drawn respectively, as shown in Figure 15 and Figure 16. It can be seen that during the task of recognition, the loss value curve of the training and verification process was well fitted, while during detection, the loss value curve of the training and verification process was well fitted in the initial stage, but with the increase in the number of iterations, the fit degree decreased and then produced a certain range of fluctuations. Figure 17 shows the loss value curve of the multi-task learning model in the process of training and verification. It can be seen that the two curves were also well fitted. Obviously, the better the fitting degree of the loss value curve during training and verification, the stronger the generalization ability of the model. It can, therefore, be asserted that a multi-task learning model can improve the generalization performance of a single task.

#### 4.3.2. Experiment 2: Baseline Model Experiment

To verify the effect of datasets on the baseline model, mainstream vulnerability detection tools (SmartCheck [29], Securify [22], and Mythril [13]) were employed as the baseline models for comparative experiments. SmartCheck [29] is a contract analysis tool based on static program analysis technology. It uses grammatical and lexical analyses of source codes and XML to describe the abstract syntax tree results after analysis. It then uses XPath [63] to check the security attributes of smart contracts on this basis to detect reentrancy, timestamp dependency, and denial of service vulnerabilities. Securify [22] is a contract static analysis tool based on symbolic abstraction that defines compliance and violation modes for each security attribute. Starting from the bytecode of the contract, Securify symbolically analyzes the dependency graph of the contract, extracts precise semantic facts, and uses these fact-matching patterns to obtain detection results. Securify can detect vulnerabilities such as reentrancy, unvalidated input, transaction order dependence (TOD), frozen Ether, unhandled exceptions, and more. Mythril [13] is a smudge analysis-based smart contract security analysis tool that integrates mixed symbol execution, smudge analysis, and control flow checking to detect vulnerabilities such as reentrancy, integer overflow, unknown calls, transaction order dependence, and timestamp dependence. As mentioned above, in this study, we selected reentrancy, arithmetic vulnerability, and contract contains unknown address vulnerability for detection; the results are summarized in Table 8.

It is evident from Table 8 that compared to the baseline model, the vulnerability detection performance of this model is improved noticeably. For arithmetic vulnerability, the precision, recall, and F1score of our model improved by 17.85%, 31.83%, and 24.91%, respectively, compared to Mythril. In regard to reentrancy detection, compared to SmartCheck, Securify, and Mythril, the precision of our model was enhanced by 28.68%, 19.46%, and 20.73%, respectively. The recall rate improved by 34.77%, 21.23%, and 26.14%, respectively, and the $F1_{score}$ improved by 31.69%, 20.3%, and 23.26%, respectively. As for unknown address vulnerability, the detection performance of this model was improved compared to the previous two vulnerabilities. However, as the baseline model does not support the detection of this type of vulnerability, the comparison data cannot be provided.

#### 4.3.3. Experiment 3: Comparison with ML Methods

ML-based smart contract vulnerability detection has been a hot topic in recent studies. Several representative ML methods were selected for comparison, including RNN [64], LSTM [36], and ABCNN (attention-based CNN [46]). The results are summarized in Table 9.

As can be seen from Table 9, based on our dataset, the performance of this model has obvious advantages over RNN, LSTM, and ABCNN. Taking ABCNN, which has the best performance among the contrast models, as an example, compared to the ABCNN model, this model improves the detection precision of three kinds of vulnerabilities, namely arithmetic vulnerability, reentrancy, and contact contains unknown address by 3.63%, 10.75%, and 5.44%, respectively, and the recall by 10%, 14.07%, and 7.29%, respectively. The $F1_{score}$ increased by 6.67%, 12.29%, and 6.29%, respectively, and the detection performance improved in all three indexes. Such promising values of these metrics confirm that the shared layer design of the model can effectively capture the code characteristics of smart contracts with vulnerability, and the model design of multi-task learning can improve the accuracy of model detection.

#### 4.3.4. Experiment 4: Ablation Experiments

To analyze the performance difference between our model and a single-task learning model, the detection performance of two sub-tasks was examined, namely detection and recognition. Taking the former task experiment as an example, the network structure was kept unchanged, the recognition task branch was frozen, the loss function of the network was canceled, and the output results of the characteristic network were directly input into the binary classification network. The recognition task experimental setup is similar.

As can be seen from Table 10 and Table 11, the performance of our model was significantly improved compared to the single-task model in terms of vulnerability detection and recognition. For example, compared to the single-task model, the precision, recall, and $F1_{score}$ of the vulnerability detection improved by 4.15%, 5.34%, and 4.74%, respectively. This model also attained high accuracy in recognizing the type of vulnerability, especially in the detection of reentrancy, with significant improvement to the indicators. The results show that knowledge sharing through multi-task learning can achieve better performance than the single-task model.

**Figure 18:**
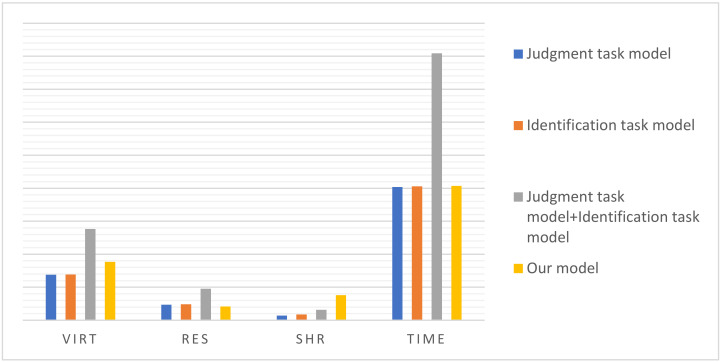
Resource usage comparison. Virtual memory usage (VIRT) indicates the total virtual memory applied for by a process, including the libraries, codes, and data used by the process, and represents the total space that the current process can access. Resident memory usage (RES) is the resident memory usage of a process; it can accurately indicate the physical memory usage of the current process by counting only the memory occupied by loaded library files. Shared memory (SHR) indicates the size of the virtual memory (VIRT) that can be shared.

To discuss the occupation of computing resources in multi-task learning, the back-end program was called to check the proportion of resources used in the single-task model experiment and the multi-task model experiment. As illustrated in Figure 18, the VIRT and SHR used by the multi-task learning model were higher than those used by the single-task model, while the RES was slightly lower. In general, although the resource memory required by multi-task learning is higher than any single task model, it is far less than the sum of resources used by the single-task model. At the same time, the multi-tasking model can simultaneously complete multiple individual tasks in approximately equal time, consuming much less time than the sum of the time consumed to complete multiple single tasks. The above data show that multi-task learning can share features among multiple tasks such that multiple tasks can be executed in parallel, thereby saving storage resources and improving execution speed at the same time.

## 5. Discussion and Conclusions

Vulnerability detection of smart contracts is an important issue for the security of blockchain. In order to improve the performance of conventional methods, a smart contract vulnerability detection model based on multi-task learning is presented. The model takes advantage of the architecture of multi-task learning, which includes a shared bottom layer and a task-specific layer. First, the opcodes of smart contracts are converted into word and position vectors by word embedding and positional embedding. They are then used in a new vector representation for successive processing. Then, a neural network based on a multi-head attention mechanism is applied to learn and extract the feature vector of the contracts. In the task layers, the classic CNN is used for the two sub-tasks of detection and recognition, which trains the features captured by the sharing layer to detect and recognize the types of vulnerabilities of contracts.

As an emergent machine learning method, multi-task learning is getting more and more attention for its high efficiency and generalization, while its application in the vulnerability detection of smart contracts is seldom reported, which makes our work valuable in this field. Moreover, the experimental results show the effectiveness of our method. In the contrast experiments, several mainstream detecting tools, such as SmartCheck, Securify, and Mythril, and some ML-based methods, such as RNN, LSTM, and ABCNN, were compared with our model. The results (Table 8 and Table 9) show that our model was superior to the contrast models in terms of detection precision. In addition, the ablation experiment results (Table 10 and Table 11) also show that our model reduced memory consumption and time consumption compared to the single-task models.

Compared to some popular detecting tools (e.g., Securify [22], SmartCheck [29], Mythril [13]), our model is based on machine learning, which can extract hidden features from massive data to improve the detection accuracy. Meanwhile, the designs of the detecting tools are usually oriented to grammatical or symbolic analysis, which limits the vulnerability types they can detect. For example, both SmartCheck and Securify are not able to detect arithmetic or the contract contains unknown address vulnerabilities, while our model can.

Compared to the common ML-based methods (e.g., RNN [64], LSTM [36], and ABCNN [46]), our model takes advantage of the architecture of multi-task learning, in which the sharing layer can learn the semantic information and extract the important feature for the processing of the task layer, helping to improve the detecting efficiency. In addition, a multi-head attention mechanism is also introduced in the sharing layer, which further enhances the efficiency.

As shown in Table 10 and Table 11, compared to the single-task model, our model had an obvious advantage in detection performance. This is because multi-task learning can improve the performance of related tasks by sharing information and complementing each other [40]. Typically, since different tasks have different local optima, the question of how to escape from them for multiple tasks is a tough issue in single-task learning, while it can be solved in multi-task learning by sharing related parts that help learn the common feature. Moreover, compared to single-task learning, such a sharing mechanism in multi-task learning saves not only the computing power but also the memory space by sharing related parts, which is also shown by the experiment results (Figure 18).

Furthermore, the vulnerability labeling work for 149,363 smart contracts collected from the XBlock platform was performed by the research group, generating a new labeled dataset that will be released soon. The dataset may be beneficial for future research in smart contract vulnerability detection.

Despite the contributions of this research, as explained above, there are still some limitations. For example, the architecture of this work adopted the hard parameter sharing method, which may weaken the expressiveness of the model and reduce the generalization ability of the model [65]. Furthermore, the weight settings of the loss function for each task are not automatically determined. In future work, further optimization of the multi-task learning architecture will be explored. For example, instead of the hard parameter sharing method, the soft parameter sharing method will be used and the shared bottom layer will be replaced by multiple hidden networks with its own parameters to improve the efficiency. In addition, some automatically weighted mechanisms for the loss function will be studied.

## Figures and Tables

**Table 1:** Attacks on smart contracts.

Attack	Types of Vulnerability	Economic Loss	Time
The DAO attack [8]	Reentrancy	Direct costs USD 60 million	June 2016
The Parity Wallet hack [9]	Code injection	Over USD 280 million were frozen	July 2017
The ERC-20 campaign [10]	Integer overflow	Indirect losses run into billions of dollars (USD)	April 2018

**Table 2:** Samples of the smart contracts datasets.

Bytecode	Flag	Arithmetic Vulnerability	Reentrancy	Contract Contains Unknown Address
0x6080604052600436100da5763fffffff7c010000…	0	0	0	0
0x606060405236156100c25763ffffffff7c0100000…	1	0	1	0
…	…	…	…	…
0x6080604052600436106101c157363fffffffc0100…	1	1	0	1

**Table 3:** The simplified opcode methods.

Substituted Opcodes	Original Opcodes
ARIT	ADD,MUL,SUB,DIV,SDIV,SMOD,MOD,ADDMOD,MULMOD,EXP,SIGNEXTEND
COMP	LT,GT,SLT,SGT
CONS1	BLOCKHASH,TIMESTAMP,NUMBER,DIFFICULTY,GASLIMIT,COINBASE
CONS2	ADDRESS,ORIGIN,CALLER
CONS3	GASPRICE,BALANCE,CALLVALUE,GAS
LOGIC	AND,OR,XOR,NOT
MEMORY	MLOAD,MSTORE,SLOAD,SSTORE,MSIZE
RETURN	RETURN,REVERT,RETURNDATASIZE,RETURNDATACOPY
PUSH	PUSH1-PUSH32
DUP	DUP1-DUP16
SWAP	SWAP1-SWAP16
LOG	LOG0-LOG4

**Table 4:** The sample data of simplified opcodes.

Opcode	Flag	Arithmetic Vulnerability	Reentrancy	Contract Contains Unknown Address
PUSH MSTORE PUSH MEMORY COMP…	0	0	0	0
PUSH MSTORE MEMORY ISZERO PUSH…	1	0	1	0
…	…	…	…	…
PUSH MSTORE MEMORY PUSH JUMPI…	1	1	0	1

**Table 5:** The number of positive and negative samples after sampling.

Type	Number		
	Before	After	Total
Arithmetic vulnerability	18,263	18,263	75,000
Reentrancy	1422	18,304	75,000
Contract contains unknown address	18,433	18,433	75,000
Non-vulnerability	104,369	20,000	75,000

**Table 6:** The experimental environment.

Software and Hardware	Configuration
Server model	Dell Precision T7920
Operating system	Ubuntu 18.04 LTS
CPU	Intel Xeon Silver 4210
GPU	NVIDIA GeForce RTX 3080
Memory size	64 GB
Disk capacity	2 T
CUDA	11.1
cuDNN	8.1.0
Python	3.7.10
PyTorch	1.14

**Table 7:** Model parameter setting.

Model Parameters	Configuration
Number of heads in multi-head attention layer	5
Number of convolution kernel windows	3
Epoch	100
Batch size	64
Learning rate	0.0001
Dropout	0.4
Optimizer	Adam

**Table 8:** Experimental results of the baseline model.

Method	Arithmetic Vulnerability			Reentrancy			Contract Contains Unknown Address		
	Precision (%)	Recall (%)	*F*1*_score_* (%)	Precision (%)	Recall (%)	*F*1*_score_* (%)	Precision (%)	Recall (%)	*F*1*_score_* (%)
SmartCheck	-	-	-	41.63	43.06	42.18	-	-	-
Securify	-	-	-	50.85	56.60	53.57	-	-	-
Mythril	59.65	52.63	55.92	49.58	51.69	50.61	-	-	-
Our model	77.50	84.46	80.83	70.31	77.83	73.87	78.85	87.94	83.14

**Table 9:** Comparative experimental results of ML methods.

Method	Arithmetic Vulnerability			Reentrancy			Contract Contains Unknown Address		
	Precision (%)	Recall (%)	*F*1*_score_* (%)	Precision (%)	Recall (%)	*F*1*_score_* (%)	Precision (%)	Recall (%)	*F*1*_score_* (%)
RNN	42.10	45.86	43.90	51.82	56.78	54.19	48.22	57.80	52.57
LSTM	44.07	57.26	49.80	51.65	67.82	58.64	52.60	57.94	55.14
ABCNN	73.87	74.46	74.16	59.56	63.76	61.58	73.41	80.65	76.85
Our model	77.50	84.46	80.83	70.31	77.83	73.87	78.85	87.94	83.14

**Table 10:** Comparison of experimental results between the judgment task model and our model.

Method	Precision (%)	Recall (%)	*F*1*_score_* (%)
Detection task model	78.02	78.77	78.39
Our model	82.17	84.11	83.13

**Table 11:** Comparison of experimental results between the identification task model and our model.

Method	Arithmetic Vulnerability			Reentrancy			Contract Contains Unknown Address		
	Precision (%)	Recall (%)	*F*1*_score_* (%)	Precision (%)	Recall (%)	*F*1*_score_* (%)	Precision (%)	Recall (%)	*F*1*_score_* (%)
Recognition task model	62.09	80.48	70.10	32.92	55.38	41.29	64.69	78.61	70.97
Our model	77.50	84.46	80.83	70.31	77.83	73.87	78.85	87.94	83.14

## Data Availability

Publicly available datasets were analyzed in this study. The data will be made available to the public soon.

## References

[1] Szabo N. (1996). Smart contracts: Building blocks for digital markets. EXTROPY J. Transhumanist Thought.

[2] Nakamoto S. (2008). Bitcoin: A peer-to-peer electronic cash system. Decentralized Bus. Rev..

[3] Dannen C. (2017). Introducing Ethereum and Solidity.

[4] Hyperledger Project. https://www.hyperledger.org/.

[5] Lin I.C., Liao T.C. (2017). A survey of blockchain security issues and challenges. Int. J. Netw. Secur..

[6] The Solidity Contract-Oriented Programming Language. https://github.com/ethereum/solidity.

[7] Atzei N., Bartoletti M., Cimoli T. A survey of attacks on ethereum smart contracts (sok). Proceedings of the International Conference on Principles of Security and Trust.

[8] Mehar M.I., Shier C.L., Giambattista A., Gong E., Fletcher G., Sanayhie R., Kim H.M., Laskowski M. (2019). Understanding a Revolutionary and Flawed Grand Experiment in Blockchain. J. Cases Inf. Technol..

[9] The Parity Wallet Hack Explained. https://blog.openzeppelin.com/on-the-parity-wallet-multisig-hack-405a8c12e8f7/.

[10] Batch Overflow Bug on Ethereum ERC20 Token Contracts and SafeMath. https://blog.matryx.ai/batch-overflow-bug-on-ethereum-erc20-token-contracts-and-safemath-f9ebcc137434.

[11] Luu L., Chu D.H., Olickel H., Saxena P., Hobor A. Making smart contracts smarter. Proceedings of the 2016 ACM SIGSAC Conference on Computer and Communications Security.

[12] Torres C.F., Schütte J., State R. Osiris: Hunting for integer bugs in ethereum smart contracts. Proceedings of the 34th Annual Computer Security Applications Conference.

[13] Mythril-Reversing and Bug Hunting Framework for the Ethereum Blockchain. https://pypi.org/project/mythril/0.8.2/.

[14] Nikolić I., Kolluri A., Sergey I., Saxena P., Hpbpr A. Finding the greedy, prodigal, and suicidal contracts at scale. Proceedings of the 34th Annual Computer Security Applications Conference.

[15] Mossberg M., Manzano F., Hennenfent E., Groce A., Grieco G., Feist J., Brunson T., Dinaburg A. Manticore: A user-friendly symbolic execution framework for binaries and smart contracts. Proceedings of the 2019 34th IEEE/ACM International Conference on Automated Software Engineering (ASE).

[16] Wood G. (2014). Ethereum: A secure decentralised generalised transaction ledger. Ethereum Proj. Yellow Pap..

[17] Formal Verification of Deed Contract in Ethereum Name Service. https://yoichihirai.com/deed.pdf.

[18] Kalra S., Goel S., Dhawan M., Sharma S. Zeus: Analyzing safety of smart contracts. Proceedings of the Network and Distributed System Symposium (NDSS).

[19] Hildenbrandt E., Saxena M., Rodrigues N., Zhu X., Daian P., Guth D., Moore B., Park D., Zhang Y., Stefanescu A. KEVM: A complete formal semantics of the ethereum virtual machine. Proceedings of the 2018 IEEE 31st Computer Security Foundations Symposium (CSF).

[20] Bhargavan K., Delignat-Lavaud A., Fournet C., Gollamudi A., Gonthier G., Kobeissi N., Kulatova N., Rastogi A., Sibut-Pinote T., Swamy N. Formal verification of smart contracts: Short paper. Proceedings of the 2016 ACM Workshop on Programming Languages and Analysis for Security.

[21] Grishchenko I., Maffei M., Schneidewind C. A semantic framework for the security analysis of ethereum smart contracts. Proceeding of the International Conference on Principles of Security and Trust.

[22] Tsankov P., Dan A., Drachsler-Cohen D., Gervais A., Bünzli F., Vechev M. Securify: Practical security analysis of smart contracts. Proceedings of the 2018 ACM SIGSAC Conference on Computer and Communications Security.

[23] Permenev A., Dimitrov D., Tsankov P., Drachsler-Cohen D., Vechev M. Verx: Safety verification of smart contracts. Proceedings of the 2020 IEEE Symposium on Security and Privacy (SP).

[24] Grieco G., Song W., Cygan A., Feist J., Groce A. Echidna: Effective, usable, and fast fuzzing for smart contracts. Proceedings of the 29th ACM SIGSOFT International Symposium on Software Testing and Analysis.

[25] Jiang B., Liu Y., Chan W.K. ContractFuzzer: Fuzzing Smart Contracts for Vulnerability Detection. Proceedings of the 33rd ACM/IEEE International Conference on Automated Software Engineering.

[26] He J., Balunović M., Ambroladze N., Tsankov P., Vechev M. Learning to fuzz from symbolic execution with application to smart contracts. Proceedings of the 2019 ACM SIGSAC Conference on Computer and Communications Security.

[27] Wüstholz V., Christakis M. Harvey: A greybox fuzzer for smart contracts. Proceedings of the 28th ACM Joint Meeting on European Software Engineering Conference and Symposium on the Foundations of Software Engineering.

[28] Zhou E., Hua S., Pi B., Sun J., Nomura Y., Yamashita K., Kurihara H. Security assurance for smart contract. Proceedings of the 2018 9th IFIP International Conference on New Technologies, Mobility and Security (NTMS).

[29] Tikhomirov S., Voskresenskaya E., Ivanitskiy I., Takhaview R., Marchenko E., Alexandrov Y. Smartcheck: Static analysis of ethereum smart contracts. Proceedings of the 1st International Workshop on Emerging Trends in Software Engineering for Blockchain.

[30] Slither. https://github.com/crytic/slither.

[31] Rodler M., Li W., Karame G.O., Davi L. Sereum: Protecting existing smart contracts against re-entrancy attacks. Proceedings of the 2019 Network and Distributed System Security Symposium.

[32] Wu S.Z., Guo T., Dong G.W. (2012). Software Vulnerability Analysis Technology. Sci. Press.

[33] Fey G. Assessing system vulnerability using formal verification techniques. Proceedings of the International Doctoral Workshop on Mathematical and Engineering Methods in Computer Science.

[34] Li J., Zhao B., Zhang C. (2018). Fuzzing: A survey. Cybersecurity.

[35] Huang T.T.H.D. (2018). Hunting the ethereum smart contract: Color-inspired inspection of potential attacks. arXiv.

[36] Tann W.J.W., Han X.J., Gupta S.S., Ong Y.-S. (2018). Towards safer smart contracts: A sequence learning approach to detecting security threats. arXiv.

[37] Wang W., Song J., Xu G., Li Y., Wang H., Su C. (2020). Contractward: Automated vulnerability detection models for ethereum smart contracts. IEEE Trans. Netw. Sci. Eng..

[38] Merity S., Keskar N.S., Socher R. (2017). Regularizing and optimizing LSTM language models. arXiv.

[39] Zhuang Y., Liu Z., Qian P., Liu Q., Wang X., He Q. Smart Contract Vulnerability Detection using Graph Neural Network. Proceedings of the International Joint Conferences on Artificial Intelligence (IJCAI).

[40] Zhang Y., Yang Q. (2021). A survey on multi-task learning. IEEE Trans. Knowl. Data Eng..

[41] LeCun Y., Bengio Y., Hinton G. (2015). Deep learning. Nature.

[42] Huang Y., Kong Q., Jia N., Chen X., Zheng Z. Recommending differentiated code to support smart contract update. Proceedings of the 2019 IEEE/ACM 27th International Conference on Program Comprehension (ICPC).

[43] Buterin V. (2014). A next-generation smart contract and decentralized application platform. White Pap..

[44] Nabilou H. (2019). How to regulate bitcoin? Decentralized regulation for a decentralized cryptocurrency. Int. J. Law Inf. Technol..

[45] Smart Contract Weakness Classification and Test Cases. http://swcregistry.io.

[46] Hirai Y. Formal verification of Deed contract in Ethereum name service. Proceedings of the 10th IFIP International Conference on New Technologies, Mobility and Security (NTMS).

[47] SeaHorn. Verification Framework. https://seahorn.github.io/.

[48] Sun Y., Gu L. (2021). Attention-based Machine Learning Model for Smart Contract Vulnerability Detection. J. Phys. Conf. Ser..

[49] Vaswani A., Shazeer N., Parmar N., Uszkoreit J., Jones L., Gomez A.N., Kaiser L., Polosukhin I. Attention is all you need. Proceedings of the 31st International Conference on Neural Information Processing Systems (NIPS).

[50] Momeni P., Wang Y., Samavi R. Machine learning model for smart contracts security analysis. Proceedings of the 2019 17th International Conference on Privacy, Security and Trust (PST).

[51] Cavnar W.B., Trenkle J.M. N-gram-based text categorization. Proceedings of the SDAIR-94, 3rd Annual Symposium on Document Analysis and Information Retrieval.

[52] Lutz O., Chen H., Fereidooni H., Sender C., Dmitrienko A., Sadeghi A.R., Koushanfar F. (2021). ESCORT: Ethereum Smart Contracts Vulnerability Detection using Deep Neural Network and Transfer Learning. arXiv.

[53] Collobert R., Weston J. A unified architecture for natural language processing: Deep neural networks with multitask learning. Proceedings of the 25th International Conference on Machine Learning.

[54] Niu J., Yang Y., Zhang S., Sun Z., Zhang W. (2019). Multi-task character-level attentional networks for medical concept normalization. Neural Processing Lett..

[55] Liu P., Qiu X., Huang X. (2016). Recurrent neural network for text classification with multi-task learning. arXiv.

[56] Yang J., Liu Y., Qian M., Guan C., Yuan X. (2019). Information Extraction from Electronic Medical Records Using Multitask Recurrent Neural Network with Contextual Word Embedding. Appl. Sci..

[57] Caruana R. (1997). Multitask learning. Mach. Learn..

[58] Duong L., Cohn T., Bird S., Cook P. Low resource dependency parsing: Cross-lingual parameter sharing in a neural network parser. Proceedings of the 53rd Annual Meeting of the Association for Computational Linguistics and the 7th International Joint Conference on Natural Language Processing.

[59] Barandela R., Sánchez J.S., García V., Ferri F.J. Learning from imbalanced sets through resampling and weighting. Proceedings of the Iberian Conference on Pattern Recognition and Image Analysis.

[60] Lin T.Y., Goyal P., Girshick R., He K., Dollar P. Focal loss for dense object detection. Proceedings of the IEEE International Conference on Computer Vision.

[61] Google Machine Learning Glossary. https://developers.google.com/machine-learning/glossary.

[62] Powers D.M.W. (2020). Evaluation: From precision, recall and F-measure to ROC, informedness, markedness and correlation. arXiv.

[63] Xpath Cover Page—W3C. https://www.w3.org/TR/xpath/all/.

[64] Goodfellow I., Bengio Y., Courville A. (2016). Deep Learning.

[65] Ma J., Zhao Z., Yi X., Hong L., Chi E.H. Modeling task relationships in multi-task learning with multi-gate mixture-of-experts. Proceedings of the 24th ACM SIGKDD International Conference on Knowledge Discovery & Data Mining.

